# The effects of viewing visual artwork on patients, staff, and visitors in healthcare settings: A scoping review

**DOI:** 10.1371/journal.pone.0328215

**Published:** 2025-08-20

**Authors:** Marcel W. Foster, Cris Sanhueza, Elisabeth Bahr, Jennifer Li-Sheen Kuo, Yaning Wu, Deborah Olaitan Komolafe, Victoria Blanchette, Tessa Brinza, Jane Morgan-Daniel, Yewande Oshodi, Kehinde Aniyat Sodimu, Nengi Omuku, Ebisan Akisanya, Larissa Trinder, Simon Willmoth, Nicola Simpson, Niamh White, Tim A. Shaw, Haley Moyse Fenning, Anna Runefelt, Mojca Kolnik, Marko Pokorn, Nils Fietje, Nisha Sajnani

**Affiliations:** 1 Jameel Arts and Health Lab, New York University, New York, United States of America; 2 Department of Music and Performing Arts Professions, New York University, New York, New York, United States of America; 3 Center for Arts in Medicine, College of the Arts, University of Florida, GainesvilleFlorida, United States of America; 4 NIHR Blood and Transplant Research Unit in Donor Health and Behaviour, University of Cambridge, Cambridge, United Kingdom; 5 Obafemi Awolowo University, Osun, Nigeria; 6 University of Florida Health Science Center Libraries, Gainesville, Florida, United States of America; 7 College of Medicine University of Lagos, Lagos, Nigeria; 8 Department of Psychiatry, Lagos University Teaching Hospital, Lagos, Nigeria; 9 The Art of Healing, Lagos, Nigeria; 10 Arts in Medicine, New York City Health + Hospitals, New York, New York, United States of America; 11 Institute for Sustainable Worlds, Norwich University of the Arts, Norwich, United Kingdom; 12 Hospital Rooms, London, United Kingdom; 13 Nordic Art Initiative, Hälsingland, Sweden; 14 Pediatricna Klinika, Univerzitetni Klinicni Center Ljubljana, Ljubljana, Slovenia; 15 Behavioural and Cultural Insights (BCI) Unit, World Health Organization Regional Office for Europe, Copenhagen, Denmark; De Montfort University, UNITED KINGDOM OF GREAT BRITAIN AND NORTHERN IRELAND

## Abstract

**Background:**

The integration of visual art in healthcare settings has been demonstrated to contribute to well-being. However, the impact of visual arts in healthcare has been primarily evaluated among patients. Viewing visual art could be a health resource to a greater number of people in healthcare settings, including patients, staff, and visitors.

**Methods:**

We conducted a scoping review to synthesize literature on the impact of viewing visual artwork among patients, staff, and visitors in healthcare settings related to the reported outcomes of well-being, wellness, and belonging. The review was informed by Arksey and O’Malley and Joanna Briggs Institute frameworks with masked pairs of reviewers. Included studies were in English, with no restrictions on geographical settings or publication dates. Nine academic databases and twelve gray literature sources were searched, in addition to a hand search and global call for submissions.

**Results:**

From an initial 25,222 records, 68 publications met inclusion criteria across 20 locations. 35 were peer-reviewed studies and 33 constituted gray literature. Included publications that reported sample sizes reflected a total of 6,006 participants with the majority being patients (3,133) followed by staff (1,343), visitors (32), and other/unspecified participants (996). Reported outcomes for patients indicated that visual arts in hospitals reduced heart rates, improved reported mental health outcomes, increased well-being, and provided a positive distraction. Reported outcomes for healthcare staff included an increased well-being, belonging, and capacity to prioritize patient needs. Reported outcomes for visitors consisted of an improved experience in healthcare environments and increased well-being.

**Conclusions:**

Our synthesis of evidence indicates that integration of visual arts within healthcare settings has positive outcomes for its viewers. Our findings are useful to promote the generation of evidence that can reliably inform the design and experience of healthcare environments.

## Introduction

Recent research has demonstrated that engagement in the arts, including visual arts, can lead to numerous health benefits [1,2]. Visual arts practices, which can include paintings, murals, ceramics, sculpture, photography, digital media, and other related media [3], play an integral role across cultures and geographical boundaries [4]. Descriptions of how participants engage with the visual arts are varied in scientific literature. Firstly, “active” participation describes processes of making or creating visual artworks in clinical [5] and non-clinical contexts [6]. Conversely, “receptive” [7,8] participation denotes viewing or observing art, and/or listening or touching for participants who are visually impaired [9].

As a construct, well-being has been understood to encapsulate psychological concepts related to physical and mental health [10–12], as well as social connectedness and engagement [12,13]. Relatedly, wellness has also been described as a psychological construct [14] cited as a possible outcome of viewing visual art [15]. While well-being and wellness are clearly overlapping concepts, efforts have been made to distinguish the two terms [16]. Given the array of efforts to define well-being [10–13,17,18] and also wellness [16], this scoping review adopts the terms as heterogeneous concepts and aims to identify literature that reflects the scope of definitions to inform future studies. Definitions for the purposes of this scoping review are included for the published protocol [19].

**Fig 1:**
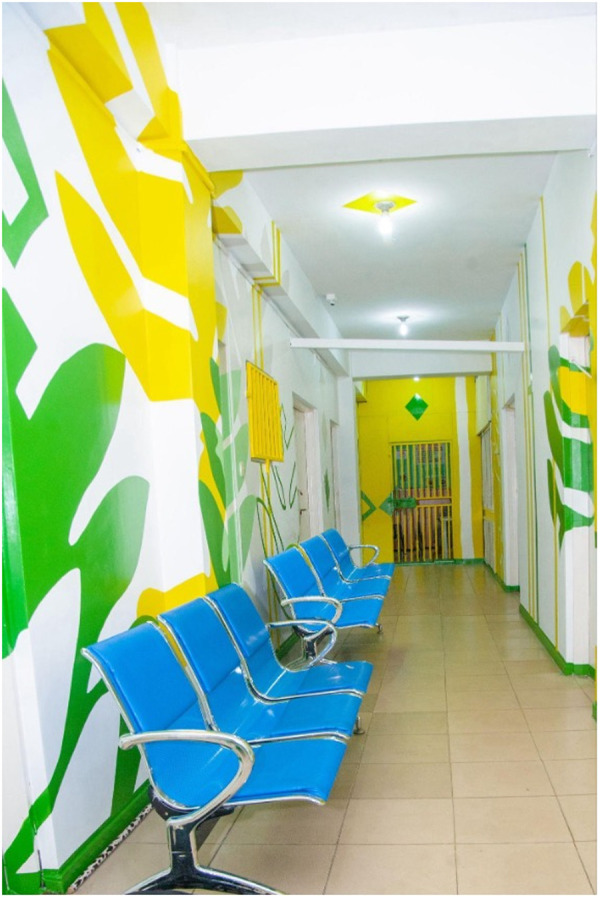
Visual art (i.e., a mural) as part of a corridor in a mental health hospital in Lagos, Nigeria. Photo courtesy of The Art of Healing.

Viewing visual art has been demonstrated to confer well-being benefits in, for example, museums [20,21], neighborhood murals [22], and online [20]. In the context of viewing visual art in healthcare settings (Fig 1), several studies that examined patients’ experiences reported outcomes related to well-being and wellness which included: visual art as a contextual factor related to patient well-being [23], an increase in the perceived quality of care [24], access to positive distractions that aided in stress reduction [25,26], reductions in anxiety [25,27,28], management of pain [29], and reductions in heart rate and systolic blood pressure among pediatric patients [30]. Evidence also suggests that in addition to patients, family members of pediatric patients experienced enhanced well-being by viewing visual art [31], and there are increasing media reports on how this viewing aids in welcoming visitors in health settings [32–34].

Healthcare staff in mental health facilities reported experiencing increased environmental satisfaction by viewing animations of natural scenes [35]. The visual design of healthcare settings, including visual art displays, was found to contribute to well-being among staff [36,37]. In addition, visual arts education has been linked to an increase in well-being among nurses [38] and improved diagnostic skills for medical students [39].

These studies strongly align with the growing evidence of workplace belonging as a vital construct for understanding well-being and wellness among healthcare personnel [40,41]. Workplace belonging refers to experiences of mattering, identification, sense of pride, goal alignment, and positive relationships at work [41–43]. Belonging has been attributed as an important factor for nurses [42,43], medical students and residents [44], and women healthcare professionals in their overall sense of workplace well-being [45]. In two cases, healthcare organizations have utilized art workshops to improve a sense of belonging among staff [37,46]. While this emerging evidence cites active engagement for visual art participation, it suggests the need to better understand existing literature related to receptive engagement and workplace belonging in healthcare settings. Workplace belonging [41] differs from general belonging [40], which encompasses a wide sense of connection and community. Workplace belonging specifically relates to the experience of feeling valued, accepted, and connected within one’s work environment [47]. Including this construct allows for a more thorough examination of belonging in various contexts.

Despite the potential of visual arts to benefit a broader population within healthcare settings, research remains limited on how incorporating visual art may collectively enhance the interrelated reported outcomes of well-being, wellness, and belonging across patients, staff, and visitors. While numerous reviews have investigated various perceptions and impacts of viewing visual art among patient-focused populations [23,25,31,36,48–52], there remains a gap in understanding how visual art may positively affect other viewers in a healthcare setting. Given the emerging evidence related to the potentially positive reported effects for visitors [31–34] and contributions to workplace belonging for staff [37,53], this review sought to expand its analysis beyond patient well-being exclusively to include patients, staff, and visitors.

### Study aims

This scoping review summarizes the literature on receptive art viewing and its impact on well-being, wellness, and belonging in the context of healthcare. We summarize key characteristics of included publications including study designs, types of settings and visual artifacts, populations engaged and present a thematic analysis of reported outcomes for patients, staff, and visitors.

## Materials and methods

The protocol for this study was registered with the Open Science Framework (OSF359ds) on February 6, 2024 [19]. The scoping review was conducted in accordance with the Joanna Briggs Institute (JBI) methodology for scoping reviews [54] and relied on Arksey and O’Malley’s framework [55] to structure analyses in six phases: 1) identifying research questions, 2) identifying relevant studies, 3) study selection, 4) charting the data, 5) collating, summarizing, and reporting the results, and 6) consultation. Consultation took place throughout the process per best practices associated with team-led scoping reviews [56]. The review used Covidence, which is aligned with Preferred Reporting Items for Systematic Reviews and Meta-Analyses extension for Scoping Reviews (PRISMA-ScR) [57], a standardized reporting guideline.

### Stage 1: Research questions

This review aimed to more fully comprehend the breadth and scope of studies related to the reported effects of well-being, wellness, and belonging when participants view visual artwork in healthcare settings. The primary research question was: what research has been conducted on the well-being, wellness, and belonging effects of viewing visual artwork in healthcare settings?

The review investigated the following research sub-questions using the PICOS framework to structure the inquiries [58], which is detailed in the inclusion and exclusion criteria below:

1. **Field(s)/Discipline(s) of Program:** In which fields and/or disciplines is the visual art program contextualized (e.g., clinical practice, public arts engagement, etc.)?
2. **Study Design(s):** What methods were used to assess the reported effects of the visual art (e.g., questionnaires, interviews, attendance tracking, arts-based methods, etc.)?
3. **Participant(s) Engaged:** Who was engaged in research involving visual arts in healthcare settings (e.g., patients, healthcare staff, visitors)?
4. **Type of Facility(ies):** In which kind of healthcare *facility* did the visual art program take place (e.g., hospital, clinic, birthing center, etc.)?
5. **Healthcare Setting(s):** In what kind of healthcare *setting* were the visual programs described (e.g., waiting room, inpatient room, lobby, etc.)?
6. **Intervention(s):** What kinds of visual artwork were described in the healthcare program (e.g., painting(s), mural(s), sculpture(s), photography, etc.)?
7. **Outcome(s):** What are the reported outcomes related to well-being, wellness, and/or belonging as a result of viewing visual art in a healthcare setting?

### Stage 2: Identifying relevant studies

A preliminary search was carried out in PubMed and Google Scholar on December 12, 2023, and the investigation was further expanded through spider webbing and citation chasing [59]. No duplicative protocols or manuscripts were located, although some reviews related to health, well-being, and the visual arts were identified and are outlined above. A health sciences librarian (JMD) developed the search strategy with research team input, based on the PICOS criteria described below. Test searching occurred in PubMed between January 18 and February 16, 2024, using the pearling technique [60] to ensure the retrieval of relevant articles known to the research team. The search strategy aimed to locate published and unpublished studies written in any language and spanning all dates; as such, no database limits or filters were employed. Truncated keywords and phrases were searched within the title and abstract fields, along with relevant subject headings adapted for each database. Following peer-review by a second health sciences librarian on February 21, 2024, the final literature searches were conducted in nine databases that were selected for their broad coverage of health and arts topics.

#### Database search.

The following databases were searched by the librarian between February 27–29, 2024 using title/abstract fields and subject headings where available: EBSCOhost’s Alt HealthWatch (1984 – Present), Art and Architecture Source (1914 – Present), CINAHL (1976 – Present), Psychology and Behavioral Sciences Collection (1930 – Present), and PsycINFO (1600 – Present); Elsevier’s Embase (1947 – Present) and Scopus (1788 – Present); PubMed; and Web of Science. Timeframes differed for each database as this review has no time restrictions, therefore, searches were set for the beginning date of each databases’ records. An updated search took place on April 24, 2025 using the same databases. In response to peer reviewer comments, seven search terms were added in the title field and as major subject headings to expand the wellbeing conceptualization, and sculpture was added as an arts keyword and subject heading. The overall number of results for the database searches was 25,222 before de-duplication and 13,232 following automated de-duplication in Covidence. An example search strategy is provided in the appendices and all search strategies are available on request (S1 File).

#### Gray literature search strategy.

A gray literature search was conducted through a manual search of web-based archives, an open call for resources, and an additional online hand search. Three reviewers (VO, CS, JK) conducted a comprehensive hand search of 12 web-based archives from February 2024 through March 2024, which included Alliance for the Arts in Research Universities (a2ru), American Art Therapy Association (AATA), American Music Therapy Association (AMTA), International Expressive Arts Therapy Association (IEATA), National Arts in Hospitals Network (UK), National Centre for Creative Health (UK), National Endowment for the Arts Research Publications (NEA), National Organization for Arts in Health (NOAH), The Culture Health and Wellbeing Alliance, University College London (UCL), University of Florida Center for Arts in Medicine Research Database, and the Wallace Foundation. Each search was documented in a Google spreadsheet, logging the date of search, archive name, number of identified materials, number of materials meeting inclusion criteria, keywords used, and relevant notes. Each document underwent a dual-review process to confirm eligibility before being uploaded to Covidence for full text review.

An open call for resources was shared on social media (X, LinkedIn, Instagram) and email to colleagues and organizations in the field from early April 2024 to mid-June 2024. Submissions were collected via a Google form. Each document underwent a dual-review process to confirm eligibility before being uploaded to Covidence for Full Text Review. Finally, four reviewers (JK, YW, RS, RD) conducted a hand search using Google in Incognito mode from late May 2024 to late June 2024. They employed targeted keywords and combinations, including “visual arts,” “murals and hospitals,” “art viewing,” and “visual arts, health settings, well-being” and recorded the search date, search terms used, types of materials, and URL of the selected materials for further screening. The purpose of using the web browser in Incognito mode was to disable the personalization that Google applies to customize results in efforts to improve the reproducibility of the search. The cache and cookies from Incognito sessions are automatically cleared when the sessions close. Each document underwent a dual-review process to confirm eligibility before being uploaded to Covidence for full text review. A final hand search was conducted and two publications were identified for inclusion.

### Stage 3: Study selection

Unique references were uploaded into the web-based software platform Covidence in preparation for screening and review. Definitions of key terms were outlined and made available to all reviewers [19]. The study selection process occurred through four phases: 1) a pilot screening of titles and abstracts in Covidence of five relevant studies identified by MF to test and confirm definitions of eligibility criteria; 2) a review of eligibility criteria completed by all reviewers; 3) the actual screening of titles and abstracts in Covidence; 4) a pilot screening of five full text identified by MF and tested in Google Forms to confirm definitions of the data extraction tool; and 5) the actual screening of full texts in Covidence. Eight co-authors (MF, CS, JK, EB, YW, TB, VO, MP) independently participated in the initial screening of titles and abstracts. Any discrepancies were addressed through discussion. Gray literature identified through the hand search phases was reviewed by four reviewers (JK, YW, RS, RD) before being uploaded into Covidence. Two reviewers (MF, EB) conducted quality checks for all included and excluded titles preceding data extraction.

#### Inclusion and exclusion criteria.

Masked pairs of researchers completed the article title and abstract screening and review of full-text papers using Covidence. The reviewers used standardized screening questions within Covidence’s data extraction tool. Inclusion criteria permitted publications in English only, with no exclusion based on when articles were published or geographical location. Each publication included in this review required agreement between two reviewers, with a third reviewer arbitrating when necessary (MF, EB, NS). The review’s inclusion criteria were carried out using the PICOS framework [59], as outlined below.

- Population (P): The participants for this scoping review include any person exposed to visual art in a healthcare setting without limitation on geography, time, or age. Healthcare setting is defined as hospitals, clinics, community health, and public health settings. Additionally, all geographies and time settings will be included in this review.
- Intervention (I): The intervention was a visual art program, intervention, or practice in which visual art products (e.g., paintings, murals, sculptures, digital media, video, etc.) were intentionally featured in a healthcare facility/setting for receptive engagement.
- Comparator (C): No comparative intervention.
- Outcome (O): All (reported) outcomes related to well-being, wellness, and belonging were included.
- Study design (S): All research designs were included.

### Stage 4: Charting the data

After identifying the full texts, the authors developed the data extraction instrument using an abductive approach [61] that sourced deductive themes identified from previous literature as well as themes inductively identified through iterative discussions in reviewing the source literature (S2 File). The instrument outlines key definitions and references that the reviewers relied on to consistently code findings for the review’s research question and seven sub-questions. The reviewers conducted a pilot extraction of three sample studies to increase the consistency and quality of the data extraction process. Masked and paired reviewers (MF, EB, CS, RS, YW, JK, VB, DK) extracted the data in accordance with the tool, and two lead reviewers (MF, EB) finalized a consensus on the extraction from the paired reviewers. Weekly quality checks were conducted by MF and EB and all included full texts were reviewed before initiating data extraction.

### Stage 5: Collating, summarizing, and reporting the results

The included studies were categorized based on the seven sub-questions for this review. The extracted data were synthesized, summarized in a tabular form using Microsoft Excel Pivot Tables, and presented in a narrative summary with accompanying graphics, qualitative insights (i.e., quotes from the publications as needed), and tables included in the Supplemental files of this manuscript.

### Stage 6: Consultation

Consultation in scoping reviews may happen throughout the process and is viewed as an essential approach to gathering contextual insights about the inquiry that may not be as visible in the literature and exchanging knowledge with potential stakeholders [56]. Consultation about this scoping review and emerging insights were discussed with stakeholders, including academics, artists, administrators, and providers from healthcare centers in the US (LT), UK (SW, NS, TS, NW, HMF), Nigeria (YO, KAS, NO), and Slovenia (AR, MK, MP). In addition, opportunities for public consultation on the role of visual artwork in hospitals were hosted by the Jameel Arts & Health Lab.

## Results

**Fig 2:**
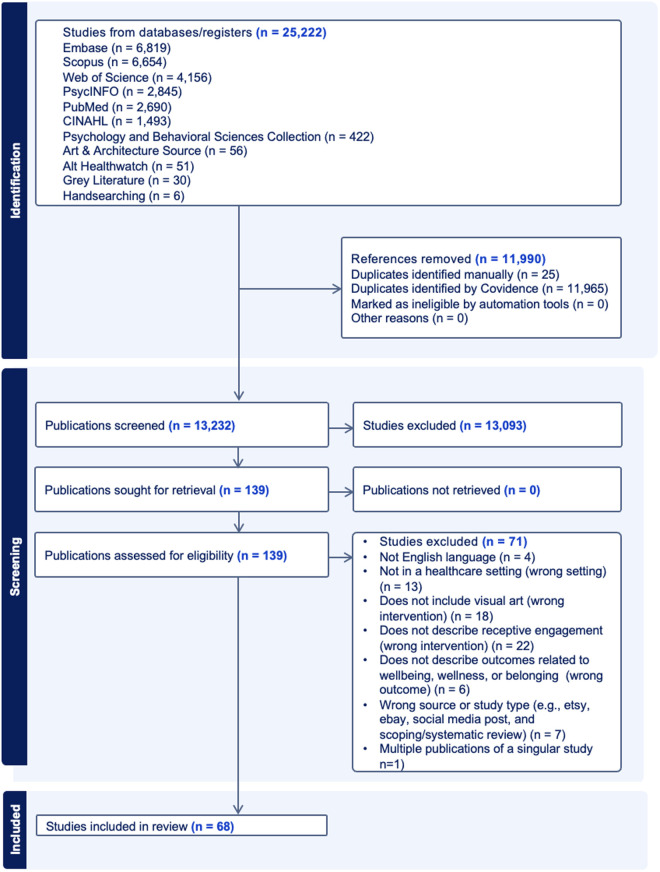
PRISMA flowchart.

Of 25,222 records, 68 publications met the inclusion criteria. 35 were from peer-reviewed sources, and 33 represented gray literature (Fig 2, Table 1). 100 images were identified across the publications and four were selected to support the findings, with permission from the authors.

**Table 1:** Key characteristics of included publications.

Peer-Reviewed Literature					
Reference Number	First Author/ Organization	Year	Document Title	Journal/Publication Title/Document Type	Location
[62]	Abulawi	2023	The conceptual design themes of artwork in the public spaces of children’s hospital	Civil Engineering and Architecture	Palestine
[63]	Bae & Asojo	2022	Interior environments in long-term care units from the theory of supportive design.	Health Environments Research & Design Journal	United States
[64]	Baumann	2013	The meaning and value of taking part in a person-centred arts programme to hospital-based stroke patients: findings from a qualitative study.	Disability & Rehabilitation	United Kingdom
[65]	Beukeboom	2012	Stress-reducing effects of real and artificial nature in a hospital waiting room.	The Journal of Alternative and Complementary Medicine	The Netherlands
[66]	Biddiss	2018	Positive distraction in pediatric healthcare waiting spaces: sharing play not germs through inclusive, hands-free interactive media	Developmental Neurorehabilitation	Canada
[67]	Bonett	2015	Ceiling art in a radiation therapy department: its effect on patient treatment experience	Journal of Medical Radiation Sciences	Australia
[68]	Butler	2020	Art and mental health in the women’s psychiatric intensive care unit	Journal of Psychiatric Intensive Care	United Kingdom
[69]	Caspari	2011	The importance of aesthetic surroundings: a study interviewing experts within different aesthetic fields.	Scandinavian Journal of Caring Sciences	Norway
[70]	Caspari	2007	Why not ask the patient? An evaluation of the aesthetic surroundings in hospitals by patients.	Quality Management in Healthcare	Norway
[71]	Csipke	2016	Design in mind: eliciting service user and frontline staff perspectives on psychiatric ward design through participatory methods.	Journal of Mental Health	United Kingdom
[72]	Dalke	2006	Colour and lighting in hospital design	Optics and Laser Technology	United Kingdom
[73]	Farrell	2016	Art research in Australian Catholic hospitals.	International Journal of Social, Political & Community Agendas in the Arts	Australia
[74]	Gao	2021	Inpatient perceptions of design characteristics related to ward environments’ restorative quality	Journal of Building Engineering	China
[75]	Gashoot	2022	Revisiting healing environments: Islamic interior elements in hospital rooms in North Africa.	Health Environments Research & Design Journal	Libya
[76]	Gore	2022	The therapeutic potential of bedside art observation in hematologic cancer inpatients: a randomized controlled pilot study.	Supportive Care in Cancer	United States
[77]	Hamed	2019	Hospital servicescape design for inpatient wellbeing	Services Marketing Quarterly	Egypt
[24]	Hill	2020	The influence of postoperative environment on patient pain and satisfaction: a randomized trial.	American Journal of Obstetrics & Gynecology	United States
[78]	Ho	2015	Art viewing directives in hospital settings effect on mood.	Health Environments Research & Design Journal	Hong Kong
[79]	Huet & Holttum	2016	Art therapy-based groups for work-related stress with staff in health and social care: An exploratory study	The Arts in Psychotherapy Research	United Kingdom
[28]	Karnik	2014	A Hospital’s Contemporary Art Collection: Effects on Patient Mood, Stress, Comfort, and Expectations	Health Environments Research & Design Journal	United States
[80]	Lone	2021	Art heals: randomized controlled study investigating the effect of a dedicated in-house art gallery on the recovery of patients after major oncologic surgery.	Annals of Surgery	United States
[27]	McCabe	2013	‘Open Window’: a randomized trial of the effect of new media art using a virtual window on quality of life in patients’ experiencing stem cell transplantation	Psychooncology	United Kingdom
Peer-Reviewed Literature					
Reference Number	First Author/ Organization	Year	Document Title	Journal/Publication Title/Document Type	Location
[81]	McCunn	2020	Impacts of large-scale interior murals on hospital employees: a pharmacy department case study	Journal of Facilities Management	Canada
[82]	Mendelson	2023	Using photographs to bring dignity to patients and help clinicians find meaning and joy in work.	Journal of Palliative Medicine	United States
[83]	Monti	2012	Pictorial intervention in a pediatric hospital environment: Effects on parental affective perception of the unit	Journal of Environmental Psychology	Italy
[84]	Mroczek	2005	Hospital design and staff perceptions: an exploratory analysis	The Health Care Manager	United States
[85]	Nanda	2011	Effect of visual art on patient anxiety and agitation in a mental health facility and implications for the business case.	Journal of Psychiatric & Mental Health Nursing	United States
[86]	Nielsen	2017	How do patients actually experience and use art in hospitals? The significance of interaction: a user-oriented experimental case study.	International Journal of Qualitative Studies on Health and Well-being	Denmark
[87]	Payam	2023	Designing well-being: a qualitative investigation of young patients’ perspectives on the material hospital environment	Health Environments Research & Design Journal	Germany
[30]	Pearson	2019	The physiological impact of window murals on pediatric patients	Health Environments Research & Design Journal	United States
[88]	Saarinen & Broxterman	2023	The impact of art on the workplace: constructing an aesthetically soothing workplace through art	Book chapter from: Workplace Wellness: From Resiliency to Suicide Prevention and Grief Management	United States
[89]	Saraiva	2022	The role of illustration in pediatric hospitalization: a collaborative project between Esad and Pedro Hispano’s Hospital of Matosinhos	Convergências	Portugal
[90]	Sui	2023	The impact of physical environments on outpatient mental health recvery: a design-oriented qualitative study of patient perspectives.	PLoS One	United States
[91]	Trevisani	2010	Art in the hospital: its impact on the feelings and emotional state of patients admitted to an internal medicine unit.	The Journal of Alternative and Complementary Medicine	Italy
[92]	Windle	2018	The impact of a visual arts program on quality of life, communication, and well-being of people living with dementia: a mixed-methods longitudinal investigation.	International Psychogeriatrics	United Kingdom
**Gray Literature**					
**Reference Number**	**First Author/ Organization**	**Year**	**Document Title**	**Journal/Publication Title/Document Type**	**Location**
[93]	Ball	2018	The art of medicine: giving meaning to art in hospital care.	Lancet -	United Kingdom
[94]	Bruce-Gordon	2023	Lifting the clouds: a work of inspiration and hope by Painting in Hospitals	Paintings in Hospitals	United Kingdom
[95]	Clementi	2019	Rep-Arte: bringing art in oncology	Tumori	Italy
[96]	Cleveland Clinic	2016	How much does a hospital art collection improve patient experience?	Cleveland Clinic	United States
[97]	Cohen	1996	Art with heart in a transitional space	Union Institute Doctoral Thesis	United States
[98]	Davies	2020	The magical painting that got me through cancer: in a lyrical testament to the healing power of art, Barbara Davies shares why she bought the skyscape that hung on her hospital wall	Daily Mail	United Kingdom
**Gray Literature**					
**Reference Number**	**First Author/ Organization**	**Year**	**Document Title**	**Journal/Publication Title/Document Type**	**Location**
[99]	Dawood	2019	Filling hospitals with art reduces patient stress, anxiety and pain	Design Week	United Kingdom
[100]	Department of Veteran Affairs	2016	Healing environment design guidelines	Department of Veteran Affairs	United States
[101]	Douglas	2011	‘The environment matters’ and ‘designing toward the whole’.	Nursing Economics	United States
[102]	Duncan	2003	A study of the effects of visual and performing arts in health care	Chelsea and Westminster Hospital	United Kingdom
[103]	Edwards	2023	The Wolfson Prize: designing the hospital of the future.	Future Healthcare Journal	United Kingdom
[104]	George	2023	The value of integrating visual arts (VIVA): evaluating the benefits of hospital room artwork on inpatient wellbeing	Penn State College of Medicine	United States
[105]	Holland	2022	Everyone seemed at ease: how art is making hospital visits less painful	The Guardian	United Kingdom
[106]	Houston Methodist	2018	West Pavilion redesign: addressing unit clinical and aesthetic goals with large-scale artwork	Houston Methodist	United States
[107]	Iwenwanne	2019	Meet the young man using art to help patients in Nigerian hospitals	TRT World	Nigeria
[108]	Johnson	2017	Creativity improves wellbeing’: art transforms mental health ward	The Guardian	United Kingdom
[109]	Jurblum	2023	Bionomic fractals and evidence-based design: improving patient and staff outcomes in an acute psychiatry ward	Australian and New Zealand Journal of Psychiatry	Australia
[110]	Kidd	2015	Exploring the use of digital picture frames on schizophrenia inpatient wards	Psychiatric Services	Canada
[111]	Knibbs	2023	Immersive hospital lights help improve mood of patients	BBC	United Kingdom
[112]	Landes	2023	Healing through art: Muscogee Nation’s Council Oak Comprehensive Healthcare is also a tribal art museum	Tulsa People	Muscogee Nation
[113]	Landro	2014	More hospitals using the healing powers of public art	Wall Street Journal	United States
[114]	Lankston	2010	Visual art in hospitals: case studies and review of the evidence.	Journal of the Royal Society of Medicine	United Kingdom
[32]	Marshall	2022	Bringing world-class art, and wonder, to mental health patients	The New York Times	United Kingdom
[115]	Mural Man	2024	Wallpaper wall murals create motivational environments in rehab facilities, hospitals and other wellness centers	Magic Murals Blog	United States
[116]	Nordic Art Initiative	2024	A friendlier hospital environment	Nordic Art Initiative Website	Slovenia
[117]	Paintings in Hospitals	2022	Art for your GP practice	Paintings in Hospitals	United Kingdom
[118]	Reed	2007	Painting Hospital Art as a Cost-effective Mental Health Program For Jails	American Jails	United States
[119]	Starlight Children’s Foundation	2022	Arts & health retrospective 2015–2022	Starlight Children’s Foundation	Australia
[120]	Stoppard	2021	What should hang on the walls of a hospital?	The New Yorker	United Kingdom
[121]	Sutter Health	2024	Sutter Health Mural	Sutter Health	United States
**Gray Literature**					
**Reference Number**	**First Author/ Organization**	**Year**	**Document Title**	**Journal/Publication Title/Document Type**	**Location**
[122]	Webb	2023	From clinical to colorful: volunteer artists install paintings throughout UNM Hospital	The University of New Mexico	United States
[123]	Wecker	2019	Fine art is good medicine: how hospitals around the world are experimenting with the healing power of art	Artnet	Spain and United States
[119]	Willmoth	2021	Arts and mental health: facing the future	Norwich University of the Arts	United Kingdom

^**a**^All peer-reviewed articles were research manuscripts. Details related to document type are included for the gray literature articles.

Materials were dated between 1996 and 2024, with 34 materials (50% of identified publications) published between 2019–2024. Included materials reflected an engagement with 6,006 participants, with peer-reviewed sources including 5,197 participants and gray literature sources including 809 participants. Three peer-reviewed sources and 26 gray literature sources did not report the number of participants. Included publications represented seven global regions and 20 locations, with the majority coming from the United States followed by the United Kingdom. Of the 33 gray literature publications included, 13 were news reports (39%), followed by seven program reports by healthcare and/or arts organizations (21%), five editorial pieces (15%), four digital media materials (e.g., blogs, art depictions) (12%), three conference proceedings (9%), and one thesis (3%). Additional information about findings is provided in the supplemental analyses (S3 File).

### Field(s)/discipline(s) of program

Among the 35 peer-reviewed studies, 23 manuscripts were presented in journals related to clinical and therapeutic practice; 13 publications contextualized the research related to the built environment (e.g., effects of aesthetic surroundings); ten were contextualized in mental health research; four were grounded in health systems and administration (e.g., how visual art programs affected operations of staff and the facility); and two articles focused on the role of arts and culture institutions in collaboration with healthcare organizations. Most studies were found to contribute to more than one field.

Among the 33 gray literature publications, the majority of documents were also grounded in discussions related to clinical and therapeutic practice with 23 of the publications relating to this field. Eight publications focused on the built environment; seven were found to focus on mental health practices and settings; seven highlighted the role of arts and culture institutions; and one publication was related to health systems and administration. The data extraction table details definitions for each field/discipline (S2 File).

### Study design(s)

Among the peer-reviewed literature, 13 studies used quantitative methods, and six studies used qualitative methods. In nine cases, mixed methods (i.e., combination of quantitative and qualitative methods) were reported, with five publications detailing mixed methods that involved arts-based methodologies. Two studies relied on physiological measures as part of their quantitative measures (e.g., heart rate), and two publications outlined methods that were analyzed as “other.”

Within the gray literature, seven publications included a detailed methodology. Two materials employed qualitative methods. Two used quantitative approaches, one of which utilized physiological and quantitative measurements. Two reports documented mixed methods exclusively, with one report using mixed methods as well as arts-based methods. The remaining 26 gray literature publications utilized informal surveys, testimonies, and/or unspecified descriptions as part of their reporting.

Twenty separate validated scales were reported across the publications. The Spielberger State-Trait Anxiety Inventory was cited in five times and the Hospital Anxiety and Depression scale was cited twice. Other scales included: Dementia Quality of Life, Herth Hope Index, Hospital Consumer Assessment of Healthcare Providers and Systems, Patient Dignity Questions, Professional Quality of Life Scale, and the Warwick-Edinburgh Mental Wellbeing Scale. A comprehensive list of additional scales is provided in Table 2.

**Table 2:** Methodological approach and measures identified across peer-reviewed and gray literature.

Study Design	Citation	Data Collection	Validated Scales
Mixed Methods	Cohen 1997 (Gray)	Questionnaires + interviews	
	Farrell 2016	Questionnaires + focus groups	
	Gao 2021	Questionnaires (used in Non-randomized Controlled Trial) + semi-structured interviews	Hospital Indoor Restoration Scale [124]
	Gore 2022	Questionnaires + observations (used in Randomized Controlled Trial)	Speilberger State Trait Anxiety Inventory [125]
	Ho 2015	Questionnaires + semi-structured interviews	Brief Mood Introspection Scale [126]
	Huet & Holttum 2016	Interviews +Quantitative self-report measure	Professional Quality of Life Scale Version [127]
	Jurblum 2023 (Gray)	Quantitative + Qualitative Measures	
	McCabe 2011	Randomized prospective clinical trial + Semi-Structured Interviews	Hospital Anxiety and Depression Scale [128] Distress Thermometer [129]
	Mendelson 2023	Questionnaires + interviews	Patient Dignity Question [130]
	Nanda 2011	Before-and-after study + interviews	
	Windle 2018	Questionnaires + interviews + standardized observation	The Greater Cincinnati Chapter Well-Being Observation Tool [131]; Dementia Quality of Life [132]; Holden Communication Scale [133]
Mixed Methods + Arts-Based Methods	Abulawi 2023	Artwork evaluation + art workshops + semi-structured interviews	
	Butler, 2019	Questionnaires + visual matrix focus group method	
	Csipke 2016	Semi-structured interviews + questionnaires + autophotographic study	
	Gashoot 2022	Qualitative consultation + computer-aided design creation	
	Payam 2023	Semi-structured interviews + draw, write, and tell method	
	Willmoth 2021 (Gray)	Questionnaires + Observation + Creative Arts Workshops	
	Bae 2022	Structured Interviews	
	Baumann 2013	Structured interviews	
Qualitative	Caspari 2011	Semi-structured interviews	
	Clementi 2019 (Gray)	Semi-structured interviews	
	Dalke 2006	Semi-structured interviews	
	Kidd 2015 (Gray)	Interviews + observation	
	Nielsen 2017	Observation	
	Sui 2023	Semi-structured interviews + observation	
Quantitative	Beukeboom 2012	Cross-over trial	Profile of Mood States; [134] Speilberger State Trait Anxiety Inventory [125]
	Biddiss 2019	Cross-over trial	Speilberger State Trait Anxiety Inventory [125]
	Bonett 2015	Questionnaires	
	Caspari 2007	Questionnaires	
	Duncan 2003 (Gray)	Physiological measures and questionnaires (used in Non-randomized Controlled Trial)	The Hospital Anxiety and Depression Scale [128]
	George 2018 (Gray)	Questionnaires	Speilberger State Trait Anxiety Inventory [125] Room Assessment survey [135]
	Hamed 2019	Questionnaires + Clinical measures (e.g., length of stay)	
	Hill 2020	Questionnaires	Hospital Consumer Assessment of Healthcare Providers and Systems [136]; Patient Global Impression of Improvement [137]
	Lone 2021	Pain measurements + questionnaires (used in Randomized Controlled Trial)	Herth Hope Index [138]; Speilberger State Trait Anxiety Inventory [125]; Warwick-Edinburgh Mental Wellbeing Scale [139]
	Karnik 2014	Questionnaires (used in Randomized Controlled Trial)	
	McCunn 2020	Questionnaires	Affective Commitment Scale [140]; Perceived Productivity Scale [141]; Perceived Restorativeness Scale [142]
	Monti 2012	Questionnaires	
	Mroczek 2005	Questionnaire	Scale of the Affective Quality Attributed to Place [143]
	Pearson 2018	Physiological measures & Questionnaires	
	Trevisani 2010	Physiological measures and length of stay	
Other	Saarinen 2023	Questionnaires	
	Saraiva 2022	First-person observations	

### Participants engaged

A total of 6,006 participants were reported across 68 publications, with 32 (of 35) peer-reviewed articles including reports on participant numbers and seven (of 33) gray literature materials including participant details (S3 File). Within peer-reviewed articles, the total number of participants was 5,197, with the minimum at 10, maximum at 826, median at 77, and the average at 162. Patients represented the most studied population with 2,689, followed by staff at 1,003, non-specified/other participants at 973, and visitors at 532. There were additionally 973 participants who were not-specified in the articles and/other categorized as other.

Tracking population engagement across the peer-reviewed publications more broadly, a total of 29 peer-reviewed studies (i.e., 83% of peer-reviewed publications) engaged patients, with 21 including adult patients, five focusing on pediatric patients (under the age of 18), and four that engaged older (i.e., geriatric) patients (over the age of 65). Thirteen studies included staff, with ten engaging healthcare staff, five that included non-healthcare staff (e.g., administrators, non-client facing pharmacists at a hospital [81]), and two with staff whose roles were not specified. Visitors were included in a total of eight studies, with only two publications engaging visitors exclusively, one of which involved volunteers for a study who had once received treatment as pediatric patients at the hospital [75]. Finally, one paper interviewed experts in healthcare aesthetics [69], one of two studies categorized as “other”.

Participant details were much more limited for gray literature. Among the seven studies that included a numerical breakdown of participants, the total number reported was 809. The number of patients reported was 444, with 340 staff reported, 23 unspecified/other participants, and two visitors.

For broader descriptions of population engagement across the 33 gray literature publications, 27 materials included descriptions about adult patients, seven included pediatric patients, and four included geriatric patients. 19 publications (58%) of these gray literature publications discussed the engagement of staff, and 13 publications (39%) mentioned visitors. Finally, two publications discussed the role of artists as well as other program stakeholders (i.e., categorized as ‘other’).

### Type of facility

More than half of peer-reviewed publications (20 studies) took place in non-specialty/general hospitals, five in pediatric hospitals, five at mental health centers, two at long term care facilities, two in a cancer center, and one in a pediatric rehabilitation center. The majority of gray literature reported on non-specialty hospitals (15 publications), six mental health centers, five pediatric hospitals, and a total of seven additional settings.

### Healthcare settings

Peer-reviewed manuscripts reported the majority of visual art works displayed in public settings with ten publications listing corridors, healthcare entrances, lounges, waiting rooms, and hospital galleries explicitly. Nine articles detailed visual art featured in more private settings such as patient and emergency rooms, and eight manuscripts included visual art programs in combined public and private settings (e.g., murals in corridors and staff areas). Finally, eight studies either did not specify the healthcare setting of the visual art or listed several possible settings (e.g., cafeteria, patient rooms, lobbies, waiting rooms, lounges, etc.). Gray literature included seven publications that explicitly mentioned public spaces, six documents that specified private spaces, and eight combined public/private spaces. Twelve publications listed multiple settings or did not specify.

### Intervention(s)

**Fig 3:**
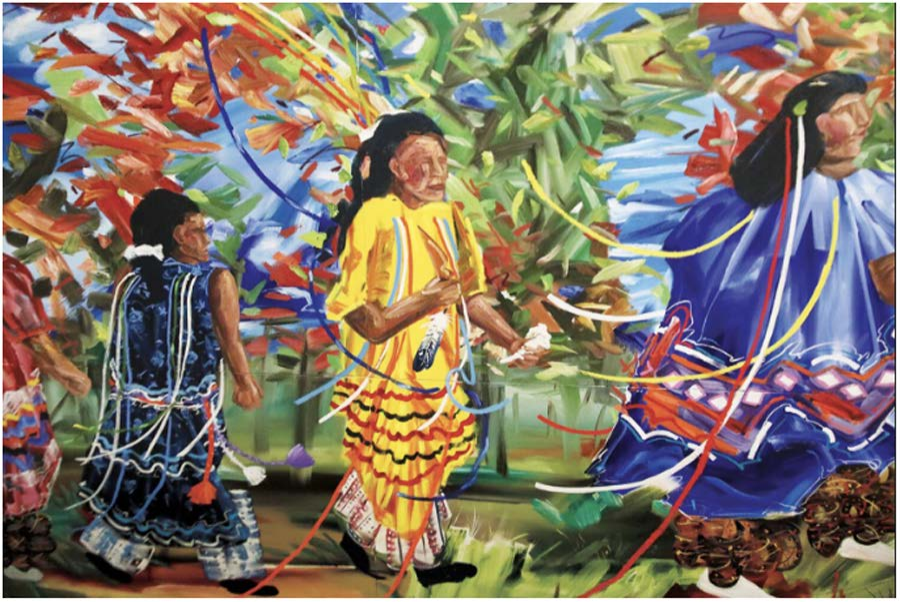
Painting from Muscogee Nation [112]. “Yatika Fields’ ‘The Ribbon Dance’ on display at Council Oak Comprehensive Healthcare.”.

In the peer-reviewed literature, four manuscripts described paintings and three described a combination of paintings along with another medium (e.g., drawings, photos, murals) (Fig 3). Four publications studied photographs exclusively, with three additional publications examining the effect of photographs combined with another visual art medium. Additionally, four articles reported on murals, three investigated digital art, and four publications described a variety of visual art forms: crafts, large projections, posters and graffiti, as well as “architectonic” forms. Six studies were categorized as “multimedia” in this review, as the publications provided at least four different visual art media employed. Finally, four separate studies did not specify the kinds of artwork included in their healthcare settings but mentioned artwork generally.

**Fig 4:**
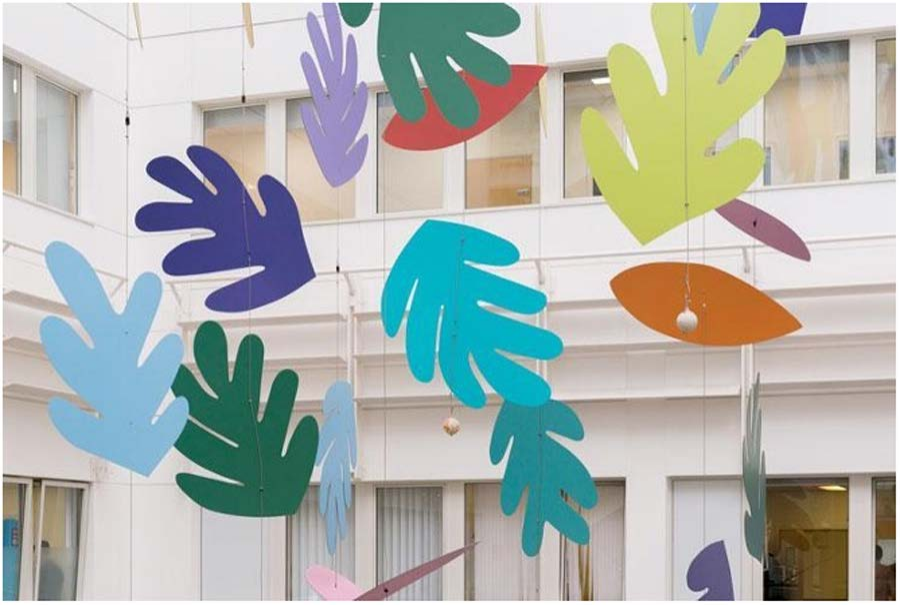
A mobile, hanging sculpture in a hospital in London, United Kingdom [99]. Atrium installation, by Sian Tucker, image courtesy of CW + .

In the gray literature, 13 publications (39%) were categorized as “multimedia” in this review, as the materials described four or more types of visual media employed (e.g., sculptures, video projection, embroidery, and general descriptions “pictures”) (Fig 4). Five publications exclusively discussed murals, three specified only paintings, and ten documents combined no more than three visual art disciplines. Finally, two documents did not specify the kind of visual art included in their healthcare setting.

### Outcomes

**Fig 5:**
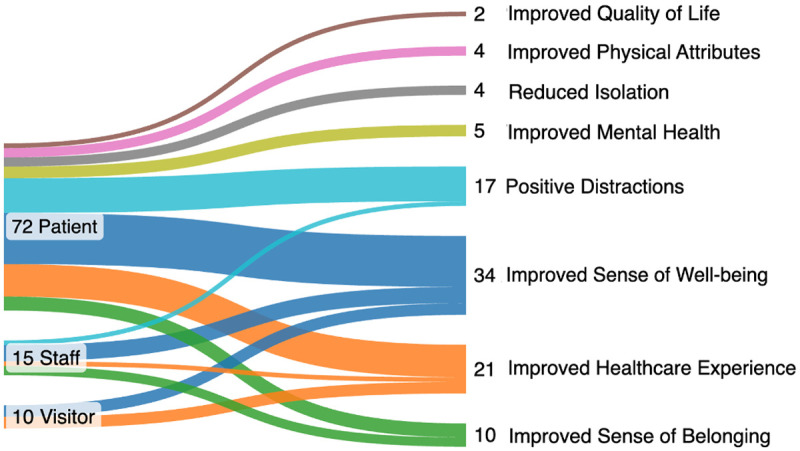
Well-being, Wellness, and Belonging Reported Outcomes Across Peer-Reviewed and Gray Literature Publications.

Reported outcomes are detailed below for peer-reviewed publications, followed by gray literature materials. The reported outcomes for gray literature were only included from the seven documents that described a detailed methodology. Across the publications, reported outcomes were found for patient, staff, and visitor populations. (Fig 5).

#### Peer reviewed literature.

**Patients.** Among the 25 peer-reviewed articles that included patient reported outcomes, 72% (18 publications) of the studies reported outcomes related to an improved sense of well-being, 52% (13 publications) found that the artwork improved healthcare experiences, and 52% (13 publications) reported that the art provided a positive distraction. One study that reported an improved experience in the healthcare setting included patients who, in addition to viewing art, were exposed to music [24].

Four studies that utilized validated mental health scales reported improved mental health outcomes. Five studies reported an increased sense of belonging, and two studies measured a reduction of perceptions of isolation. Three studies were categorized in this review as improving physical attributes for patients, which included reduced heart rates [30] as well as reported improvements in sleep quality [72]. Finally, two studies reported an improved quality of life.

Six studies reported some negative effects of receptive engagement with visual arts on reported outcomes [28,64,65,73,85,92]. Finally, five articles that used quantitative measures reported some null findings, meaning that predictions on pain reduction [24,80], quality of life [92], decreased blood pressure [30], and questions related to perceptions of healthcare quality [68] were found to be statistically insignificant.

**Staff.** In the peer-reviewed literature, six studies reported outcomes for staff participants. The reported outcomes included an improved sense of well-being [28,68,72,79,82], sense of belonging [68,72,79,88], as well as three qualitative reports that included negative perceptions of the artworks [67,78,85]. Three quantitative studies found that the artworks had no kind of measured effect for staff [79,81,84].

**Visitors.** Six peer-reviewed studies included visitors as participants, and four of these studies found that the visual art contributed to a sense of well-being [62,72,73,75]. Additionally, five manuscripts reported that the art influenced a higher perception of quality for the healthcare setting. No negative and/or null findings were reported for visitor participants.

#### Gray literature.

**Patients.** Patient-reported outcomes were included in five documents. These reported outcomes were enhanced emotional well-being, art as a positive distraction, an improved sense of health experience and belonging, and in one instance reduced physical discomfort, which was included as an improved physical attribute in Fig 5. A study reported statistically insignificant findings related to its predictions on quality of life, anxiety reduction, and self-reported pain among patients [104]. A separate publication also reported some negative findings from interviews, with patients noting preferences and dislikes for various paintings displayed [95].

**Staff.** Three documents reported outcomes for staff and found that the art aided with a sense of workplace belonging, well-being, and capacity to prioritize patients’ needs (i.e., coded as Improved Healthcare Experience in Fig 5). One publication included an insight with a negative quote, describing an image as “too busy” [97].

**Visitors.** One article reported outcomes related to visitors found that the art contributed to a sense of well-being. Finally, one publication combined its art intervention in a healthcare setting along with live music [102], therefore, the findings from this document cannot be linked to the visual art exclusively.

## Discussion

### Overview

The findings of this review suggest that visual art has been demonstrated to contribute to a range of interrelated reported outcomes for people who spend time in healthcare settings, with the majority of included publications referring to well-being. As noted in other studies [2,22,53], the ubiquity, accessibility, and impact of visual art on affect, cognition, perception, and social interaction make it a viable, yet likely underutilized, health resource.

### Facilities and settings of the visual art

In general, the majority of the visual art exhibitions across the identified literature took place in general hospitals. Within the peer-reviewed articles, this was often a result of either not specifying the setting and/or looking across multiple healthcare sites [70,72,73]. A substantive proportion of the literature identified also took place in healthcare settings designed for pediatric patients as well as elderly adults (i.e., geriatric patients). Researchers have recognized that childhood and older age are two life stages where interventions to boost psychosocial well-being are especially salient. For youth, patients expressed fear related to sterile spaces and visual art interventions have been historically cited to address these concerns [31]. Likewise for elderly adults, arts engagement has been discussed as a dignified strategy for engaging in meaningful communications, particularly for people who are experiencing memory loss [144,145].

The settings of the artworks (e.g., inpatient private rooms vs. more “public” settings like hallways/waiting rooms) were fairly equal across peer-reviewed and the gray literature. The objective of the document (e.g., research aim or purpose of the article) often determined the population and the contextual surroundings of what the participant viewed. For private/inpatient settings, the visual art was often cited as a strategy to create a positive distraction for patients [24,27,82]; whereas for settings in more “public” environments (e.g., corridors, foyer, waiting rooms) focused on outcomes related to holistic well-being for patients, visitors, and staff [72,78,88,102]. In one paper, the parents of pediatric patients (i.e., visitors) were surveyed to understand how murals impacted their experience as well as perceptions on its effects on their patient children [83].

The fact that this review did not identify a particular setting that was most common across the literature suggests the ubiquity and diverse utility of art in hospitals. Indeed, visual art was found on ceilings, floor-tiling suggestions, and the exterior of buildings.

#### Methodological insights.

Research on the reported use of visual art to positively affect patients, staff, and visitors increased between 2019–2024 with significant methodological variance in how the impact of viewing visual art was studied.

Quantitative measures were reported in 31 (i.e., included in mixed methods) of the 42 publications that included a detailed methodology. The Speilberger State-Trait Anxiety Inventory was cited in five of these studies, which aligns with reported outcomes related to well-being and receptively engaging with visual art [65,66,76,80,104]. Three publications used physiological measures (e.g., heart rate, systolic blood pressure) to assess reported outcomes that may be valuable to include in future research to build on existing evidence related to neuropsychological models of experiencing the visual arts [146,147]. While these quantitative findings are promising, the heterogeneity presented across the study designs merits further analysis to establish groundwork for a core outcome set related to the experience of visual art in hospitals and healthcare settings.

Qualitative studies presented opportunities to hear directly from people with lived experience in healthcare settings. For example, in a qualitative study that explicitly asked in its title, “How do patients actually experience and use art in hospitals,” [86] one participant stated that “*… (The art) provides safety… you feel yourself shielded in a way.”; and “I think I would be more relaxed (if there had been something on the wall), instead of this white wall…*” [86]. Relatedly, in a qualitative study of an intervention where long-term care patients were invited to bring photographs to view and share with their healthcare workers, a thematic analysis identified increased joy for patients: “*It keeps my spirits alive. That’s for sure. It gives me hope. It gives me joy*” [82].

**Fig 6:**
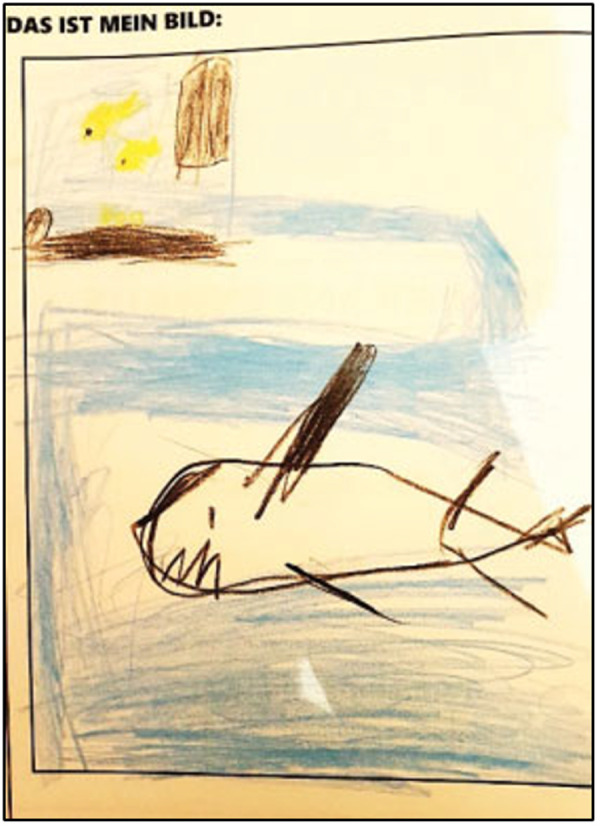
Drawing by a youth participant to inform future public spaces at a children’s hospital in Munich, Germany [87].

Similarly, the inclusion of arts-based methods which, while only used in six of the included publications [62,68,71,75,87,119] demonstrated the value of artistic methods (e.g., photography, drawing) in eliciting participating experiences. In four cases, arts-based methods were used to inform the current and/or future designs of the hospital facility [62,71,75,87]. Two studies focused on pediatric settings [62,87], and in one instance, included images as part of their findings with recommendations from youth informants to incorporate images of fish on the floor of the lobby to suggest an aquatic environment (Fig 6) [87].

### Reported outcomes

**Patient Reported Outcomes.** The majority of reported outcomes were specific to patients, with a strong emphasis on youth [30,62,87,89,97], as well as publications pertaining to elder adults [28,64,92]. Additionally, two gray literature publications described engagement with veteran populations [97,100] and a third involved a correctional setting [118]; however, methodological details were not provided, and therefore, reported outcomes could not be included in this review. In addition to outcomes related to an “improved sense of well-being,” an “improved healthcare experience” was reported 21 times for all participant types, and “positive distractions” were reported 17 times for patients as well staff; these three outcomes represented the majority of reported effects. Given the heterogeneous definitions of well-being [11–13,17], wide usage of the term across the literature comes as no surprise. “Improved healthcare experience” has been addressed in a wide range of literature as well, with a systematic review outlining studies that documented how visual aspects in a hospital were strongly associated with positive patient outcomes [148]. And when considering the outcome of “positive distraction,” this specific construct has been addressed in other studies [149,150], and the literature cited in this review linked the distractions with physiological outcomes [30], emotional relief [86], and social connectedness [27].

**Fig 7:**
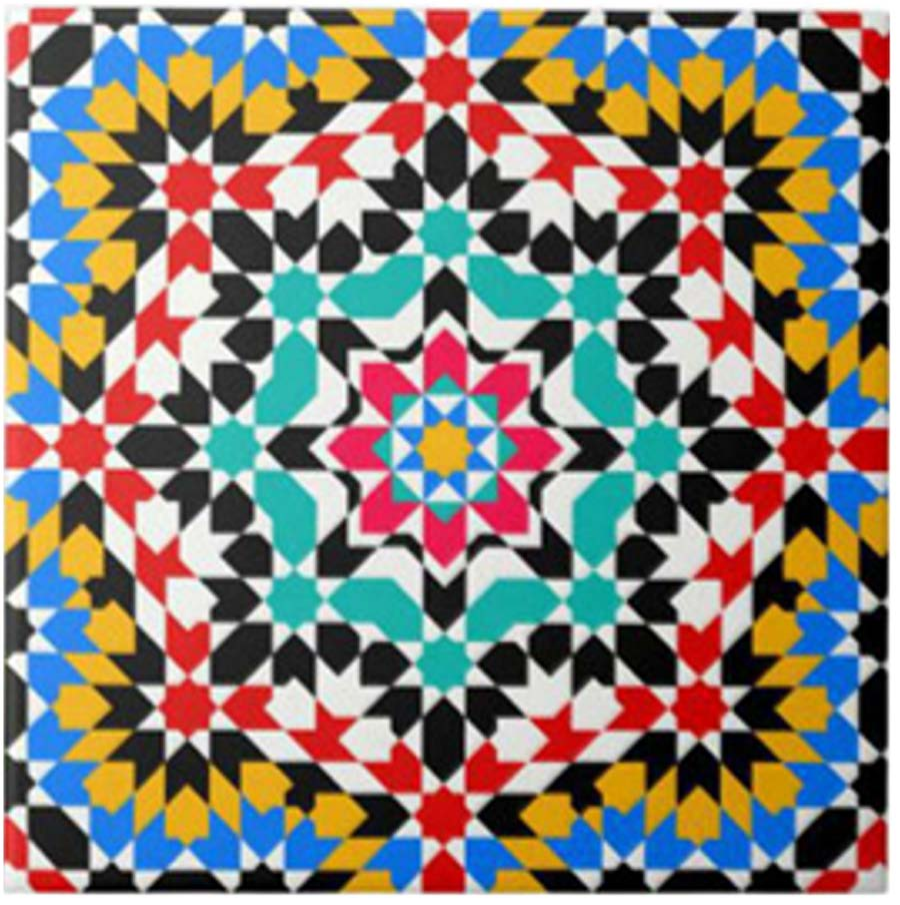
Islamic-inspired art and design influenced the hospital aesthetics in a hospital in Tripoli, Libya [75]. Example of Islamic botanic patterns.

**Staff Reported Outcomes.** Workplace belonging was included and used for all participant populations as an additional construct to capture the correct breadth of literature related to the research goal. The lack of studies on staff well-being, wellness, and belonging was noteworthy, especially when considering the epidemic of burnout in healthcare settings [151]. Finally, while this review did not focus on architectural contributions to well-being explicitly, there is evidence that the experience of space and the built environment is tied to healthcare staff well-being [152]. To this end, two studies attributed well-being outcomes to the spiritual epistemologies that influenced the design of healthcare spaces, including the imagery used in Islamic design in Egypt (Fig 7) [77] and across Catholic hospitals in Australia [153]. Cultural considerations were highlighted as necessary to the interpretation of visual arts in healthcare settings in numerous studies [62,75,82,87,100,112].

**Outcomes Reported Across Reviews.** While there was great variance in the purposes and reported outcomes related to viewing visual art, findings were largely consistent with other reviews that focused on related research aims. Reported reductions in heart rate and decreased symptoms of anxiety were also found in other evidence syntheses [25,36]. In a systematic review of the impact of viewing art on well-being in any kind of setting, findings from empirical research suggested that receptive engagement correlated with constructs of eudemonic well-being [154]. For reviews that specified outcomes related to patients, virtual reality [23] as well as paintings that featured biophilic and/or identifiable figures [52] were found to be associated with well-being outcomes. These findings are consistent with those from this review. Two reviews also examined the effects of viewing visual art for non-patients and reported well-being outcomes for visitors (i.e., parents of pediatric patients) [31] and staff [36]. In both cases, these reviews did not distinguish active vs. receptive engagement. Our findings are consistent with these reviews in that outcomes related to anxiety reduction and improved healthcare experience were found for visitors and staff.

### Negative and null findings

No publication reported an adverse event as a result of viewing visual art. Any negative and/or statistically insignificant finding was consistently presented with other reported outcomes that were positive (i.e., aligned to hypotheses that viewing the visual art would align with reported outcomes of well-being, wellness, and/or belonging). In one case, viewing a nature video in a waiting room among pediatric patients increased measures of anxiety [66]. However, this intervention focused on positive distractions and included positive findings for engaging with other media, such as handheld digital devices and aquariums. All other negative findings were related to qualitative observations related to a participant’s dislike of an artwork. For example: “*The artwork looks like an afterthought and does not seem very relevant to the patient experience*” [71].

Nine articles that detailed statistical methods reported null findings related to hypotheses that viewing visual art would correlate with measures of well-being and/or belonging. For patients, viewing visual art was not found to reduce reports of pain [24,80,104], or experiences of depression/distress [27]. For pediatric patients who were found to have decreased heartrates related to viewing visual art, these same patients did not report reduced systolic blood pressure [30]. Similarly, art programs for dementia patients showed initial stimulation but no sustained improvements in quality of life or communication [92], highlighting challenges in measuring subjective well-being, particularly in populations with cognitive impairment. Finally, in an effort to understand how murals impacted psychiatric patients, several constructs related to patient experience yielded statistically insignificant findings [68] (i.e., hope that the care would help them; perceptions of staff kindness, etc.).

Null findings for staff included a survey on building environment with insignificant results related to viewing art [84] and a visual art viewing workshop where employees mostly reported insignificant results related to lessening workplace stress [79]. Additionally, an initiative that placed biophilic murals (e.g., large photographs of forest landscapes) in a hospital basement for pharmaceutical staff that found the images made no difference in psychosocial outcomes, (including the lack of enhancement in well-being, commitment, productivity, or attention restoration) compared to their control setting [81]. Contextual factors, such as mural placement (narrow corridor wall) and limited staff involvement in the commissioning process, also may have influenced staff responses. The authors reflected that *“...organizations ought to ensure that occupants understand links between the alteration and their experience with the physical setting,”* [81] meaning that engaging patients, staff, and visitors in the making or relating to visual arts may serve to amplify the intended benefits. Indeed, the processes that give rise to the choices made about which visual arts to incorporate in healthcare settings are important, if not more so than the actual content included in the final image itself [53].

Challenges related to long-term data collection, including the lack of pre-installation data [81] and reliance on retrospective questions that may not accurately reflect shifts in employee perceptions [84], as well as homogeneous study samples [24], may have hindered the detection of significant changes. The variability and complexity of responses based on individual differences and contextual factors was a shared null finding, with both patients and staff demonstrating inconsistent improvements in experiences following visual art interventions [81,84,92].

## Strengths and limitations of this study

A strength of this study is the focus on all constituents in healthcare settings as well as inclusion of well-being outcomes, including both wellness and belonging, which have salience in a healthcare context. Another potential strength was the inclusion and rigorous analysis of gray literature. By synthesizing the content of news reports, blog posts, and other media, a more diverse understanding of the research question was presented and early evidence was included in this more nascent topic in health sciences. This consideration is important for the inclusion of arts and health programs in historically underrepresented global regions where resources to support peer-reviewed studies may be limited. Several limitations must be considered. Publication bias emphasizing statistically significant and/or positive findings may have biased the findings reported in the included literature. The majority of identified publications were from arts in healthcare programs based in either the United States or the United Kingdom; this is partially since publications eligible for review were limited to English. The databases queried for gray literature were not comprehensive, and several archives were likely omitted. Finally, as a critical appraisal was not conducted, the findings must therefore be considered conservatively.

## Directions for future research

The challenges related to what precisely constitutes visual art have been widely discussed [5,21], and this study intentionally included a broad range of cultural practices and artifacts. In this study, visual art was defined as any kind of artifact that was exhibited programmatically for the purpose of receptive engagement and, therefore, included personal photographs brought in by patients [82], architectural interventions to enhance hospital design experience [69], color and lighting design efforts [72], and other diverse media. Given the array of visual artifacts in this review, future primary studies would do well to limit the variance of visual artwork being studied. While the eligibility criteria allowed for publications related to receptive engagement of art among visually impaired participants [9], no publications were identified that described this kind of intervention. Future studies could contribute to the evidence base for the impact of active or receptive engagement with visual art on participants with visual impairments in healthcare settings. Additionally, as almost 40% (i.e., 13 out of 33) of the gray literature identified through our hand search yielded news reports, a future study looking exclusively at this topic from news database sources could yield important insights. Moreover, considering the diversity of visual art, broad scope of the literature, and varied presence of art across healthcare settings, future systematic reviews could benefit by focusing on a specified setting to better understand the contextual effects.

Similarly, the difficulty of defining well-being was cited in many studies [13,21]. Future analyses on well-being would benefit from additional theoretical modeling to examine the convergence of well-being, wellness, and belonging as constructs [146]. This would aid in efforts to identify a core outcome set for future research on visual arts in healthcare settings and related measures. Our findings also suggest that future reviews prioritizing patient experiences could build on the array of specific domains of well-being and wellness constructs, such as improved physical attributes, reduced isolation, and/or an increased sense of belonging. Additionally, mapping participants in a consistent way was also a challenge across the varied studies, and this was most notable for healthcare students, who were mentioned in three studies [71,78,95] and included as healthcare staff in the findings. Relatedly, given existing reviews including visitors did not distinguish between active and receptive engagement, future reviews would benefit from not only including non-patient participants but also specifying the type of engagement with the art.

Given the call for cultural considerations to be included in the analysis of visual elements in healthcare environments [86], future studies might consider a comparative analysis of viewing visual arts in different country contexts as well as implementation studies to examine reported outcomes in varied contexts. Future reviews could expand upon these data by systematically querying design-based interventions in healthcare settings, staff satisfaction, and aesthetic experience [147]. Given healthcare staff are the people who reside in these facilities for the longest time, it is vital to examine staff experience and cost-effective interventions that can be put in place to support their experience and effectiveness alongside that of patients and visitors. Finally, only three studies discussed the role of artists in healthcare settings; their role in facilitating the experience of visual art in these settings would also merit further investigation.

## Conclusion

This scoping review examined published literature pertaining to the effects of viewing visual art on patients, staff, and visitors in healthcare settings. The findings indicate that the inclusion of visual art may be an accessible means of optimizing healthcare environments and experiences for patients, staff, and visitors.

## Supporting information

S1 File Search string. (PDF)

S2 File Data extraction tool. (XLSX)

S3 File Supplemental analyses. (XLSX)
