# Correction: The effects of viewing visual artwork on patients, staff, and visitors in healthcare settings: A scoping review

**DOI:** 10.1371/journal.pone.0347506

**Published:** 2026-04-16

**Authors:** Marcel W. Foster, Cris Sanhueza, Elisabeth Bahr, Jennifer Li-Sheen Kuo, Yaning Wu, Deborah Olaitan Komolafe, Victoria Blanchette, Tessa Brinza, Jane Morgan-Daniel, Yewande Oshodi, Kehinde Aniyat Sodimu, Nengi Omuku, Ebisan Akisanya, Larissa Trinder, Simon Willmoth, Nicola Simpson, Niamh White, Tim A. Shaw, Haley Moyse Fenning, Anna Runefelt, Mojca Kolnik, Marko Pokorn, Nils Fietje, Nisha Sajnani

In the Results subsection of the Abstract, there is an error in the third sentence. The correct sentence is: Included publications that reported sample sizes reflected a total of 6,006 participants with the majority being patients (3,133) followed by staff (1,343), visitors (534), and other/unspecified participants (996).
