# Supplementary material for: The effects of viewing visual artwork on patients, staff, and visitors in healthcare settings: A scoping review
Source: PLoS One. 2025 Aug 20;20(8):e0328215. doi: 10.1371/journal.pone.0328215 (PMC12367177; doi:10.1371/journal.pone.0328215)
Supplement: S1 File — (PDF) [file pone.0328215.s001.pdf]

PubMed Sample Search Strategy, February 29, 2024 with April 24, 2025 included

Set Number	Search Terms
1	((art[tiab] OR arts[tiab] OR artist*[tiab] OR artwork*[tiab] OR artmaking[tiab] OR "art-based"[tiab] OR "arts-based"[tiab] OR artpiece*[tiab] OR mural*[tiab] OR paint*[tiab] OR photo*[tiab] OR drawing*[tiab] OR gallery[tiab] OR galleries[tiab] OR museum*[tiab] OR print[tiab] OR prints[tiab] OR printmak*[tiab] OR poster[tiab] OR posters[tiab] OR picture*[tiab] OR pictorial[tiab] OR video*[tiab] OR film[tiab] OR films[tiab] OR filming[tiab] OR architecture[tiab] OR craft*[tiab] OR handicraft*[tiab] OR fashion[tiab] OR quilt*[tiab] OR silkscreen*[tiab] OR "silk screen*"[tiab] OR animat*[tiab] OR caricatur*[tiab] OR cartoon*[tiab] OR clay[tiab] OR collag*[tiab] OR comic[tiab] OR comics[tiab] OR comicbook*[tiab] OR fotonovela*[tiab] OR photonovella*[tiab] OR mandala*[tiab] OR photovoice[tiab] OR "photo voice"[tiab] OR portrait*[tiab] OR potter*[tiab] OR textile*[tiab] OR watercolor*[tiab] OR watercolour*[tiab] OR "water-color*"[tiab] OR "water-colour*"[tiab] OR mosaic*[tiab] OR montage[tiab] OR montages[tiab] OR graffiti[tiab] OR sketch*[tiab] OR coloring[tiab] OR colouring[tiab] OR video*[tiab] OR movie*[tiab] OR webcast*[tiab] OR "motion picture*"[tiab] OR cinema*[tiab] OR "digital culture"[tiab] OR sew[tiab] OR sews[tiab] OR sewing[tiab] OR weave[tiab] OR weaves[tiab] OR weaving[tiab] OR crochet*[tiab] OR knit[tiab] OR knits[tiab] OR knitting[tiab] OR spinning[tiab] OR needlework*[tiab] OR needlepoint*[tiab] OR macram*[tiab] OR embroider*[tiab] OR "rug hooking"[tiab] OR tapestr*[tiab] OR dyeing[tiab] OR "tie-dy*"[tiab] OR temari[tiab] OR shibori[tiab] OR screenprint*[tiab] OR “screen print*”[tiab] OR illustration*[tiab] OR "stained glass"[tiab] OR batik*[tiab] OR lithograph*[tiab] OR decorat*[tiab] OR "visual expression*"[tiab] OR "digital media"[tiab] OR projection[tiab] OR projections[tiab] OR sculpt*[tiab] OR "Art"[Mesh:NoExp] OR "Caricatures as Topic"[Mesh] OR "Engraving and Engravings"[Mesh] OR "Medicine in the Arts"[Mesh] OR "Motion Pictures"[Mesh] OR "Paintings"[Mesh] OR "Pictorial Works as Topic"[Mesh] OR "Portraits as Topic"[Mesh] OR "Science in the Arts"[Mesh] OR "Art Therapy"[Mesh] OR "Sculpture"[Mesh]) NOT (antiretroviral[tiab] OR "anti retroviral"[tiab] OR "anti-retroviral"[tiab] OR "HAART"[tiab] OR "assisted reproductive therap*"[tiab] OR "Antiretroviral Therapy, Highly Active"[Mesh] OR "Reproductive Techniques, Assisted"[Mesh])
2	("healthcare facilit*"[tiab] OR "health care facilit*"[tiab] OR "health facilit*"[tiab] OR "medical facilit*"[tiab] OR "medical environment*"[tiab] OR "medical center*"[tiab] OR "medical centre*"[tiab] OR "health center*"[tiab] OR "health centre*"[tiab] OR "healthcare center*"[tiab] OR "healthcare centre*"[tiab] OR "health care center*"[tiab] OR "health care centre*"[tiab] OR hospital*[tiab] OR inpatient*[tiab] OR "in-patient*"[tiab] OR clinic[tiab] OR clinics[tiab] OR clinical[tiab] OR "waiting room*"[tiab] OR surgery[tiab] OR surgeries[tiab] OR surgicent*[tiab] OR outpatient*[tiab] OR "out-patient*"[tiab] OR "rehabilitation center*"[tiab] OR "rehabilitation centre*"[tiab] OR "rehab center*"[tiab] OR "rehab centre*"[tiab] OR "operating room"[tiab] OR "operating rooms"[tiab] OR "operating theat*"[tiab] OR "patient room"[tiab] OR "patient rooms"[tiab] OR "patients room"[tiab] OR "patients rooms"[tiab] OR "patient's room"[tiab] OR "patient’s rooms"[tiab] OR "patients’ room"[tiab] OR "patients' rooms"[tiab] OR ward[tiab] OR wards[tiab] OR "doctor office*"[tiab] OR "doctors office*"[tiab] OR "doctor's office*"[tiab] OR "doctors' office*"[tiab] OR "physician office*"[tiab] OR "physicians office*"[tiab] OR "physician's office*"[tiab] OR "physicians' office*"[tiab] OR "medical office*"[tiab] OR "health science center*"[tiab] OR "health science centre*"[tiab] OR "health sciences center*"[tiab] OR "health sciences centre*"[tiab] OR "medical school*"[tiab] OR "nursing school*"[tiab] OR "allied health school*"[tiab] OR "health professions school*"[tiab] OR "urgent care center*"[tiab] OR "urgent care centre*"[tiab] OR "urgent care facilit*"[tiab] OR "community health centre*"[tiab] OR "community health center*"[tiab] OR

	"community health facilit*"[tiab] OR "neighborhood health cent*"[tiab] OR "neighbourhood health cent*"[tiab] OR "health education center*"[tiab] OR "health education centre*"[tiab] OR "Health Facilities"[Mesh:NoExp] OR "Academic Medical Centers"[Mesh] OR "Ambulatory Care Facilities"[Mesh:NoExp] OR "Community Health Centers"[Mesh:NoExp] OR "Outpatient Clinics, Hospital"[Mesh:NoExp] OR "Surgicenters"[Mesh] OR "Facility Design and Construction"[Mesh] OR "Health Facilities, Proprietary"[Mesh] OR "Health Facility Administration"[Mesh] OR "Health Facility Environment"[Mesh] OR "Health Facility Moving"[Mesh] OR "Health Facility Size"[Mesh] OR "Hospital Units"[Mesh] OR "Hospitals"[Mesh] OR "Medical Office Buildings"[Mesh] OR "Physicians' Offices"[Mesh] OR "Student Run Clinic"[Mesh] OR "Rehabilitation Centers"[Mesh:NoExp])
3	(visual[tiab] OR view*[tiab] OR look[tiab] OR looks[tiab] OR looking[tiab] OR looked[tiab] OR engag*[tiab] OR tour[tiab] OR tours[tiab] OR toured[tiab] OR touring[tiab] OR appreciation[tiab] OR gallery[tiab] OR galleries[tiab] OR museum*[tiab] OR exhibit*[tiab] OR listen*[tiab] OR watch*[tiab] OR attend*[tiab] OR consume*[tiab] OR consuming[tiab] OR observ*[tiab] OR experienc*[tiab] OR interact*[tiab] OR audience*[tiab] OR "receptive art"[tiab] OR "receptive arts"[tiab] OR participat*[tiab] OR "Museums"[Mesh] OR "Exhibitions as Topic"[Mesh])
4	(wellbeing[tiab] OR "well-being"[tiab] OR "well being"[tiab] OR wellness[tiab] OR belong[tiab] OR belongs[tiab] OR belonging[tiab] OR “life satisfaction”[ti] OR happiness[ti] OR “meaning in life”[ti] OR “physical health”[ti] OR “social connectedness”[ti] OR “social engagement”[ti] OR “mental health”[ti] OR "Psychological Well-Being"[Mesh] OR "Mental Health"[Majr] OR "Happiness"[Majr])
5	(outcome*[tiab] OR effect*[tiab] OR impact*[tiab] OR response*[tiab] OR "cost effective"[tiab] OR "cost effectiveness"[tiab] OR evaluat*[tiab] OR satisfaction[tiab] OR satisfied[tiab] OR satisfy[tiab] OR dissatisfaction[tiab] OR dissatisfy[tiab] OR dissatisfied[tiab] OR burden*[tiab] OR "Outcome and Process Assessment, Health Care"[Mesh] OR "Evaluation Studies as Topic"[Mesh] OR "Personal Satisfaction"[Mesh] OR "Job Satisfaction"[Mesh] OR "Caregiver Burden"[Mesh])
6	# 1 AND #2 AND #3 AND #4 AND #5
7	#6, Filters: None

The [ti] keywords and the MeSH Major Topics in Set 4 were added in response to peer reviewer comments to expand the original search.
